# Subarachnoid Haemorrhage Incidence Pattern Analysis with Circular Statistics

**DOI:** 10.1155/2024/6631990

**Published:** 2024-04-16

**Authors:** Ashish Dravid, Wen-Shan Sung, Jeeuk Song, Arvind Dubey, Behzad Eftekhar

**Affiliations:** ^1^Department of Neurosurgery, Nepean Hospital, The University of Sydney, Sydney, Australia; ^2^Department of Neurosurgery, Royal Hobart Hospital, Hobart, Australia; ^3^Department of Neurosurgery, Australian School of Advanced Medicine, Macquarie University, Sydney, Australia

## Abstract

Knowledge about biological rhythms of diseases may not only help in understanding the pathophysiology of diseases but can also help health service policy makers and emergency department directors to allocate resources efficiently. Aneurysmal subarachnoid haemorrhage (SAH) has high rates of morbidity and mortality. The incidence of SAH has been attributed to patient-related factors such as characteristics of aneurysms, smoking, and hypertension. There are studies showing that the incidence of aneurysmal SAH appears to behave in periodic fashions over long time periods. However, there are inconsistencies in the literature regarding the impact of chronobiological factors such as circadian, seasonal, and lunar cycle factors on the occurrence of SAH. In this study, we focused on the analysis of a temporal pattern of SAH (infradian rhythms) with a novel approach using circular statistical methods. We aimed to see whether there is a circular pattern for the occurrence of SAH at all and if so, whether it can be related to known temporal patterns based on available literature. Our study did not support the notion that aneurysmal subarachnoid haemorrhages occur on any specific day in a cycle with specific lengths up to 365 days including specific weekdays, full moon, equinoxes, and solstices. Hence, we found no relationship between SAH incidence and timing. Study in larger populations using similar circular statistical methods is suggested.

## 1. Introduction

Biological rhythms are cyclic phenomena that help living organisms to adapt to different periodicities like solar or lunar rhythms. The biological rhythms are classified into (a) circadian (from the Latin circa dies, period of ∼24 hours), (b) ultradian (period <24 hours), and (c) infradian (period >24 hours) rhythms [1]. Several studies have pointed to health risks associated with desynchronized biological rhythms, including cancer, heart disease, digestive disorders, and menstrual irregularities [2]. Knowledge about biological rhythms of diseases may not only help in understanding the pathophysiology of diseases but can also help health service policy makers and emergency department directors to allocate resources efficiently.

Among all cerebrovascular pathologies, aneurysmal subarachnoid haemorrhage (SAH) represents 1–7% of all strokes [3] and has high rates of morbidity and mortality [4]. The incidence of SAH due to rupture of aneurysms may be attributed to genetic components, but it is likely to be largely affected by potential modifiable and/or predictable extrinsic factors [5].

Characteristics of the aneurysm (i.e., size [6–8], morphology [6, 9, 10], location [6, 10], and haemodynamic features [11–13]) have been consistently shown to be related to the risks of aneurysm rupture and subarachnoid haemorrhage. Studies have also supported patient-related factors such as smoking, hypertension [14, 15], alcohol [14], and physical activity [15, 16] as contributors to aneurysm rupture risk. There are studies showing that the incidence of aneurysmal SAH appears to behave in periodic fashions over long time periods [17]. However, there are inconsistencies in the literature regarding the impact of circadian [15, 18, 19], seasonal [18–30], and lunar cycle [31–34] on the occurrence of SAH.

All previous studies have investigated specific periodicities, for example, weekly, monthly, seasonal, or annual cycles. In this study, we focused on the analysis of a temporal pattern (infradian rhythms—cycles longer than one day) of SAH with a novel approach using circular statistical methods. Firstly, we aim to see whether there is a circular pattern for the occurrence of SAH at all and if so, whether it can be related to known temporal patterns based on available literature.

## 2. Methods

We extracted dates of admission under the diagnosis of SAH to Royal Hobart Hospital in Tasmania (Australia) from 8/1/1986 to 9/5/2003 from previously collected data. The dates of incidences of SAH had been extracted from medical records, following the patient privacy and data protection regulations. Ethics approval was not required for this study as the study did not involve human participation or use of any patient's personal data.

Firstly, we aimed to see whether there is a circular pattern (with cycle lengths from 1 to 365 days) for the occurrence of SAH at all. The statistical methods that deal with circular/directional data are referred to as circular statistical methods and require input data in angular format (degrees or radians). Dates of SAH incidences were converted to serial numbers (January 1, 1900, as serial number 1) using Microsoft Excel functions. The serial numbers (linear values) were then transformed to angular values measured on a scale of 0–359 degrees for each cycle length from 1 to 365 (days of a year). For example, for a four-day cycle, any serial number modulus 4 which equaled to two was transformed to 180 degrees. In order to investigate whether there is a circular pattern in our data, we needed statistical procedures to test the null hypothesis that points are spread uniformly around the circle without a preferred direction. Raleigh and Kuiper statistical tests are common circular statistical tests for uniformity. The readers are referred to related literature for further technical information about these tests [35]. Circular statistics analyses including Raleigh and Kuiper tests were performed using circstat module [36] in STATA (StataCorp. 2021. Stata Statistical Software: Release 17. College Station, TX: StataCorp LLC.).

Cycles whose both Raleigh and Kuiper test results were statistically significant (*p* ≤ 0.05) were selected. Raleigh and Kuiper test results with *p* values <0.05 are considered statistically significant implying that the incidences of SAH are likely not to be distributed uniformly. Cycle lengths that were multiples of other significant cycle lengths were removed, as if a cycle length test result is statistically significant, all multiples of that cycle length are expected to be statistically significant as well. For example, if cycle length of seven was selected, all cycle lengths that were multiples of seven (14, 21, 28,…) were removed. Among the remaining cycle lengths, we focused on those whose lengths that were within the range of known cycle lengths such as weekly or lunar cycles.

PyEphem astronomy library for Python (version 4.1.4) was used to calculate the lunar phases, equinoxes, and solstices during the period of study [37].

Python codes (version 3.11.1) were used for manipulation of data. STATA MP 17 (StataCorp. 2021. Stata Statistical Software: Release 17. College Station, TX: StataCorp LLC.) was used for descriptive analysis and Pearson chi-square test.

## 3. Results

There were 453 days in the study period that the incidence of SAH was recorded. Cycle lengths of 4, 7, 31, 53, 83, 86, 165, 167, 225, 226, 227, and 230 had both statistically significant Raleigh and Kuiper tests and thus provided evidence to reject uniformity (Table 1). That simply meant that we could not rule out that occurrence of SAH could happen uniformly in cycles with any of the abovementioned cycle lengths. As the next step, we investigated each of those cycle lengths that have been considered in the literature as biological rhythms for humans [38]. As is seen in Table 1, three known cycles could fall within the 95% confidence interval range of the selected cycle lengths: weekly cycle (7 days), lunar cycle (29.53056 days long (29 d 12 h 44 m)), and 90 and 182 days (period between equinoxes and/or solstices). We included weekly cycle as it appears that our seven-day week, which is found in many ancient and modern civilizations including the three main monotheistic religions, may be an adaptation to an endogenous biologic rhythm rather than the rhythm being a societally impressed phenomenon [39].

Each of the abovementioned cycles was further studied using chi-square tests.

There were no statistically significant differences between the days of the week (Table 2). None of the SAH occurrences exactly coincided with full moon phases, equinoxes, or solstices during the period of study.

Due to the relatively small number of cases during the study period, in addition to the exact dates of full moon phases, equinoxes, and solstices, the number of SAH incidences was calculated in equal periods before and after each of these days. The length of periods was arbitrarily selected as 1/4th of the lunar phases and 1/6th of the times between the equinoxes or solstices. For example, a lunar month was divided into four equal phases.

The number of SAH incidences was not significantly different in any of the abovementioned periods compared with other periods statistically (Tables 3–5).

The calculated range of lunar phases was 29.27–29.80 days, the range of period between vernal and autumnal equinoxes was 178.8–186.4 days, and that between summer and winter solstices was 181.7–183.5 days.

## 4. Discussion

Circular analysis is commonly used in biological sciences, in assessment of directional (e.g., animal orientation) data to time-dependent data (e.g., annual cycles and circadian rhythms). The most common statistical exploration of circular data involves testing the null hypothesis that the data are uniformly distributed across all possible values around the circle using the Rayleigh test [40]. There is no consensus in the related published literature regarding the temporal pattern of SAH. For instance, while correlation between seasonal variation and the onset of SAH was limited to certain age groups in one study [18], its correlation was largely inconsistent within and between countries [18–29]. Few studies reported higher incidence of SAH in winter/spring [18, 20, 23–25], but other studies, even from the same countries, reported no seasonal variation in the SAH occurrence [15, 19, 20, 22, 25, 26, 41].

In addition, a potential association between lunar cycle and incidence of SAH still remains elusive following inconsistent findings both within and between countries [31–33].

This generalizes further to stroke where there remain inconsistencies in the impact of chronobiological factors on different stroke types. Studies have shown that there is a relationship between moon phase and stroke, although this varied among classification of stroke [42–46]. However, others have found no difference [47].

While there are studies reporting a weekly large peak on Mondays [48], other studies that have investigated a circaseptan variation in SAH onset did not find apparent weekly patterns [18, 24, 49].

Our study did not show any temporal patterns for occurrence of SAH for cycle length of 1 to 365. In other words, this study did not support the idea that SAH occurs more often on any particular day in different cycle lengths up to 365 days.

While inconsistencies between studies could be related to the different sample size, heterogeneous populations, or potential other extrinsic factors (i.e., environmental and geographic conditions), our results could be considered as another evidence that there is no actual temporal pattern (infradian rhythms) per se in occurrence of SAH. Studies with larger sample size using similar circular statistical methods could further clarify the matter.

All previous studies have investigated specific periodicities, for example, weekly, monthly, seasonal, or annual cycles. One of the novel aspects of this study is that we examined all possible cycle lengths between 1 and 365 days. The method used in the study has not been used previously in neurological disorders and can be considered as one of the advantages of this study.

## 5. Limitations

Our study is retrospective and relies upon hospital records as there is no official registry for all SAH cases in Tasmania. A possible concern about our findings is the reliability of the data source and missing cases of SAH. Royal Hobart Hospital is the only neurosurgical referral center in Tasmania. Additionally, the incidence of SAH in our study (6 per 100000 person-years) has not been statistically different from the reported estimate of incidences in Australia (mean 5.5, range 5.3–6.0 cases per 100,000 person-years). These pieces of information make it less likely that a significant number of SAH episodes occurring in Tasmania could have been excluded from our study.

This study explores only unimodal departure from uniformity; however, there might be more than one cluster around the circle or multimodal patterns of temporal distribution of SAHs.

We used days as unit of possible cycles as the recorded timing of occurrence of SAH (hours and minutes) was not available or reliable and was therefore not explored. Even though we explored arbitrary periods of time around the full moon, equinoxes, and solstices, we mainly tried to find out if there is any specific day in a cycle with specific length, when SAH occurs more frequently. This study has not specifically explored unimodal patterns of SAH occurrences with clusters longer than one day in different length of cycles or cycle lengths longer than 365 days, which could be considered as a limitation of the study.

## 6. Conclusion

Our study did not support the notion that aneurysmal subarachnoid haemorrhages occur on any specific day in a cycle with specific lengths up to 365 days including specific weekdays, full moon, equinoxes, and solstices. Hence, we found no relationship between SAH incidence and timing. The findings are consistent with some of the related published literature. Study in larger populations using similar circular statistical methods is suggested.

## Figures and Tables

**Table 1:** Cycle lengths that had both statistically significant Raleigh and Kuiper tests.

Cycle length	95% CI lower limit	95% CI upper limit	Rayleigh	Kuiper	Known cycles could fall within the 95% confidence interval
4	3.47	4.53	0.035	0	
7	6.09	7.91	0.03	0	Weekly cycle
31	26.33	35.67	0.05	0.004	Lunar cycle
53	45.3	60.7	0.044	0.008	
83	70.9	95.1	0.05	0.039	Period between equinoxes and/or solstices
86	74.93	97.07	0.026	0.006	Period between equinoxes and/or solstices
165	102.51	227.49	0.018	0.039	Period between equinoxes and/or solstices
167	147.08	186.92	0.018	0.006	Period between equinoxes and/or solstices
225	192.94	257.06	0.038	0.014	
226	196.02	255.98	0.027	0.013	
227	141.75	312.25	0.02	0.044	Period between equinoxes and/or solstices
230	143.94	316.06	0.022	0.043	Period between equinoxes and/or solstices

**Table 2:** Distribution of incidences based on weekdays (Pearson chi2 (6) = 11.7969, Pr = 0.067).

Weekdays	Freq.	Percent	Cum.
Friday	79	17.44	17.44
Monday	63	13.91	31.35
Saturday	56	12.36	43.71
Sunday	54	11.92	55.63
Thursday	72	15.89	71.52
Tuesday	52	11.48	83
Wednesday	77	17	100
Total	453	100	

**Table 3:** Distribution of SAH incidences based of calculated lunar phases (Pearson chi2 (3) = 6.0464, Pr = 0.109).

	Freq.	Percent	Cum.
1	125	27.59	27.59
2	113	24.94	52.54
3	92	20.31	72.85
4	123	27.15	100
Total	453	100	

**Table 4:** Distribution of SAH incidences in equal periods before and after each of the equinoxes.

	Freq.	Percent	Cum.
1	72	15.89	15.89
2	78	17.22	33.11
3	62	13.69	46.8
4	77	17	63.8
5	88	19.43	83.22
6	76	16.78	100
Total	453	100	

The length of periods was arbitrarily selected as 1/6th of the times between the equinoxes (Pearson chi2 (5) = 4.7616, Pr = 0.446).

**Table 5:** Distribution of SAH incidences in equal periods before and after each of the solstices.

	Freq.	Percent	Cum.
1	82	18.1	18.1
2	77	17	35.1
3	77	17	52.1
4	69	15.23	67.33
5	73	16.11	83.44
6	75	16.56	100
Total	453	100	

The length of periods was arbitrarily selected as 1/6th of the times between the solstices (Pearson chi2 (5) = 1.2649, Pr = 0.939).

## Data Availability

Data are available upon reasonable request.

## References

[1] Cappadona R., Di Simone E., De Giorgi A., Zucchi B., Fabbian F., Manfredini R. (2021). Biological rhythms, health, and gender-specific differences. *Journal of Sex-and Gender-Specific Medicine*.

[2] Hastings M. H., Reddy A. B., Maywood E. S. (2003). A clockwork web: circadian timing in brain and periphery, in health and disease. *Nature Reviews Neuroscience*.

[3] Feigin V. L., Lawes C. M., Bennett D. A., Anderson C. S. (2003). Stroke epidemiology: a review of population-based studies of incidence, prevalence, and case-fatality in the late 20th century. *The Lancet Neurology*.

[4] Hop J. W., Rinkel G. J., Algra A., Van Gijn J. (1997). Case-fatality rates and functional outcome after subarachnoid hemorrhage: a systematic review. *Stroke*.

[5] Ruigrok Y. M., Buskens E., Rinkel G. J. (2001). Attributable risk of common and rare determinants of subarachnoid hemorrhage. *Stroke*.

[6] Lall R. R., Eddleman C. S., Bendok B. R., Batjer H. H. (2009). Unruptured intracranial aneurysms and the assessment of rupture risk based on anatomical and morphological factors: sifting through the sands of data. *Neurosurgical Focus*.

[7] Nishioka H., Torner J. C., Graf C. J., Kassell N. F., Sahs A. L., Goettler L. C. (1984). Cooperative study of intracranial aneurysms and subarachnoid hemorrhage: a long-term prognostic study: II. Ruptured intracranial aneurysms managed conservatively. *Archives of Neurology*.

[8] White P., Wardlaw J. (2003). Unruptured intracranial aneurysms-Detection and management. *Risk*.

[9] Weir B., Amidei C., Kongable G. (2003). The aspect ratio (dome/neck) of ruptured and unruptured aneurysms. *Journal of Neurosurgery*.

[10] Hademenos G., Massoud T., Turjman F., Sayre J. (1998). Anatomical and morphological factors correlating with rupture of intracranial aneurysms in patients referred for endovascular treatment. *Neuroradiology*.

[11] Cebral J. R., Mut F., Weir J., Putman C. M. (2011). Association of hemodynamic characteristics and cerebral aneurysm rupture. *American Journal of Neuroradiology*.

[12] Hassan T., Timofeev E. V., Saito T. (2005). A proposed parent vessel geometry—based categorization of saccular intracranial aneurysms: computational flow dynamics analysis of the risk factors for lesion rupture. *Journal of Neurosurgery*.

[13] Sforza D. M., Putman C. M., Cebral J. R. (2009). Hemodynamics of cerebral aneurysms. *Annual Review of Fluid Mechanics*.

[14] Feigin V. L., Rinkel G. J., Lawes C. M. (2005). Risk factors for subarachnoid hemorrhage: an updated systematic review of epidemiological studies. *Stroke*.

[15] Matsuda M., Watanabe K., Saito A., Matsumura K.-I., Ichikawa M. (2007). Circumstances, activities, and events precipitating aneurysmal subarachnoid hemorrhage. *Journal of Stroke and Cerebrovascular Diseases*.

[16] Reynolds M. R., Willie J. T., Zipfel G. J., Dacey R. G. (2011). Sexual intercourse and cerebral aneurysmal rupture: potential mechanisms and precipitants: a review. *Journal of Neurosurgery*.

[17] Rosenbaum B. P., Weil R. J. (2014). Aneurysmal subarachnoid hemorrhage: relationship to solar activity in the United States, 1988–2010. *Astrobiology*.

[18] Feigin V. L., Anderson C. S., Anderson N. E., Broad J. B., Pledger M. J., Bonita R. (2001). Is there a temporal pattern in the occurrence of subarachnoid hemorrhage in the southern hemisphere? Pooled data from 3 large, population-based incidence studies in Australasia, 1981 to 1997. *Stroke*.

[19] Nyquist P., Brown R. D., Wiebers D., Crowson C., O’Fallon W. (2001). Circadian and seasonal occurrence of subarachnoid and intracerebral hemorrhage. *Neurology*.

[20] Hakan T., Kizilkilic O., Adaletli I., Karabagli H., Kocer N., Islak C. (2003). Is there any seasonal influence in spontaneous bleeding of intracranial aneurysm and and/or AVM in Istanbul?. *Swiss Medical Weekly*.

[21] Inagawa T., Takechi A., Yahara K. (2000). Primary intracerebral and aneurysmal subarachnoid hemorrhage in Izumo city, Japan. Part 1: incidence and seasonal and diurnal variations. *Journal of Neurosurgery*.

[22] Wang Y., Levi C. R., Attia J. R., D’Este C. A., Spratt N., Fisher J. (2003). Seasonal variation in stroke in the Hunter Region, Australia: a 5-year hospital-based study, 1995-2000. *Stroke*.

[23] Fischer T., Johnsen S. P., Pedersen L., Gaist D., Sørensen H. T., Rothman K. J. (2004). Seasonal variation in hospitalization and case fatality of subarachnoid hemorrhage–a nationwide Danish study on 9,367 patients. *Neuroepidemiology*.

[24] Beseoglu K., Hänggi D., Stummer W., Steiger H.-J. (2008). Dependence of subarachnoid hemorrhage on climate conditions: a systematic meteorological analysis from the Düsseldorf metropolitan area. *Neurosurgery*.

[25] Muroi C., Yonekawa Y., Khan N., Rousson V., Keller E. (2004). Seasonal variations in hospital admissions due to aneurysmal subarachnoid haemorrhage in the state of Zurich, Switzerland. *Acta Neurochirurgica*.

[26] Oyoshi T., Nakayama M., Kuratsu J.-I. (1999). Relationship between aneurysmal subarachnoid hemorrhage and climatic conditions in the subtropical region, Amami-Oshima, in Japan. *Neurologia Medico-Chirurgica*.

[27] Cowperthwaite M. C., Burnett M. G. (2011). The association between weather and spontaneous subarachnoid hemorrhage: an analysis of 155 US hospitals. *Neurosurgery*.

[28] McDonald R. J., McDonald J., Bida J., Kallmes D., Cloft H. (2012). Subarachnoid hemorrhage incidence in the United States does not vary with season or temperature. *American Journal of Neuroradiology*.

[29] Schievink W., Wijdicks E., Meyer F., Piepgras D., Fode N., Whisnant J. (1995). Seasons, snow, and subarachnoid hemorrhage: lack of association in Rochester, Minnesota. *Journal of Neurosurgery*.

[30] Wu Y., Tang N., Xia L. (2022). Chronobiological patterns of aneurysmal subarachnoid hemorrhage in Central China. *Global Heart*.

[31] Ali Y., Rahme R., Matar N. (2008). Impact of the lunar cycle on the incidence of intracranial aneurysm rupture: myth or reality?. *Clinical Neurology and Neurosurgery*.

[32] Kamp M. A., Dibué M., Slotty P., Steiger H.-J., Hänggi D. (2013). Impact of the moon on cerebral aneurysm rupture. *Acta Neurochirurgica*.

[33] Lahner D., Marhold F., Gruber A., Schramm W. (2009). Impact of the lunar cycle on the incidence of aneurysmal subarachnoid haemorrhage: myth or reality?. *Clinical Neurology and Neurosurgery*.

[34] Banfield J. C., Abdolell M., Shankar J. S. (2017). Secular pattern of aneurismal rupture with the lunar cycle and season. *Interventional Neuroradiology*.

[35] Landler L., Ruxton G. D., Malkemper E. P. (2018). Circular data in biology: advice for effectively implementing statistical procedures. *Behavioral Ecology and Sociobiology*.

[36] Cox N. (1998). *CIRCSTAT: Stata Modules to Calculate Circular Statistics*.

[37] Rhodes B. C. (2011). Pyephem: astronomical ephemeris for python. *Astrophysics Source Code Library*.

[38] Refinetti R. (2008). Biological rhythms. *Encyclopedia of Ecology*.

[39] Haus E. (2007). Chronobiology in the endocrine system. *Advanced Drug Delivery Reviews*.

[40] Rayleigh L. (1919). On the problem of random vibrations, and of random flights in one, two, or three dimensions. *The London, Edinburgh and Dublin Philosophical Magazine and Journal of Science*.

[41] Han M. H., Kim J., Choi K. S. (2017). Monthly variations in aneurysmal subarachnoid hemorrhage incidence and mortality: correlation with weather and pollution. *PLoS One*.

[42] Ahmad F., Quinn T. J., Dawson J., Walters M. (2008). A link between lunar phase and medically unexplained stroke symptoms: an unearthly influence?. *Journal of Psychosomatic Research*.

[43] Mao Y., Schnytzer Y., Busija L., Churilov L., Davis S., Yan B. (2015). MOONSTROKE: lunar patterns of stroke occurrence combined with circadian and seasonal rhythmicity—a hospital based study. *Chronobiology International*.

[44] Wang R. R., Hao Y., Chen J. (2020). Sex differences in the effects of the moon on ischemic stroke incidence: new findings from Beijing, China. *Chronobiology International*.

[45] Lanska D. J., Hoffmann R. G. (1999). Seasonal variation in stroke mortality rates. *Neurology*.

[46] Zaręba K., Lasek-Bal A., Student S. (2021). The influence of selected meteorological factors on the prevalence and course of stroke. *Medicina*.

[47] Ruuskanen J. O., Sipilä J. O., Rautava P., Kytö V. (2018). No association of moon phase with stroke occurrence. *Chronobiology International*.

[48] Izumihara A. (2012). Epidemiology of subarachnoid hemorrhage in the Yaeyama Islands, an isolated subtropical region of Japan most frequently affected by typhoons: a population-based study. *Clinical Neurology and Neurosurgery*.

[49] Umemura K., Hirashima Y., Kurimoto M. (2008). Involvement of meteorological factors and sex in the occurrence of subarachnoid hemorrhage in Japan. *Neurologia Medico-Chirurgica*.

[50] Huang H., Lai L. T. (2020). Incidence and case-fatality of aneurysmal subarachnoid hemorrhage in Australia, 2008–2018. *World Neurosurgery*.

